# A machine learning approach for diagnostic and prognostic predictions, key risk factors and interactions

**DOI:** 10.1007/s10742-024-00324-7

**Published:** 2024-03-18

**Authors:** Murtaza Nasir, Nichalin S. Summerfield, Stephanie Carreiro, Dan Berlowitz, Asil Oztekin

**Affiliations:** 1 Finance, Real Estate, & Decision Science Department, Barton School of Business, Wichita State University, Wichita, KS 67260, USA; 2 Operations & Information Systems Department, Manning School of Business, University of Massachusetts Lowell, Lowell, MA 01854, USA; 3 Department of Emergency Medicine, University of Massachusetts Medical School & UMass Memorial Healthcare, Worcester, MA 01655, USA; 4 Department of Public Health, Zuckerberg College of Health Sciences, University of Massachusetts Lowell, Lowell, MA 01854, USA

**Keywords:** Healthcare, Machine learning, Business analytics, Decision support systems, Health service

## Abstract

Machine learning (ML) has the potential to revolutionize healthcare, allowing healthcare providers to improve patient-care planning, resource planning and utilization. Furthermore, identifying key-risk-factors and interaction-effects can help service-providers and decision-makers to institute better policies and procedures. This study used COVID-19 electronic health record (EHR) data to predict five crucial outcomes: positive-test, ventilation, death, hospitalization days, and ICU days. Our models achieved high accuracy and precision, with AUC values of 91.6%, 99.1%, and 97.5% for the first three outcomes, and MAE of 0.752 and 0.257 days for the last two outcomes. We also identified interaction effects, such as high bicarbonate in arterial blood being associated with longer hospitalization in middleaged patients. Our models are embedded in a prototype of an online decision support tool that can be used by healthcare providers to make more informed decisions.

## 1 Introduction

The use of machine learning (ML) for diagnostic and prognostic predictions in healthcare is a rapidly growing field. Crucially, the ability to predict adverse events following surgery based on patients’ presurgical clinical data, such as electronic health record (EHR) data, holds paramount significance. Such predictions empower both physicians and patients to make informed decisions, enhancing the overall decision support process. In recent years, the surge in clinical data availability and advancements in computing power have fueled remarkable progress in ML techniques, enabling the extraction of valuable insights from these rich clinical datasets. In particular, ML algorithms have emerged as key components of assisted medical decision-making procedures, offering the potential for accurate diagnostic and prognostic predictions by leveraging EHRs.

The core challenge in addressing this task lies in formulating an appropriate function f that effectively maps each input data point X to the desired output y, denoted as y=f(X). Notably, clinical datasets present unique complexities due to their vast size and diverse data types, encompassing diagnoses, treatments, vital signs, and laboratory values. These mixed data types further compound the intricacies of the ML problem, necessitating the development of sophisticated algorithms capable of handling such complexities with precision and accuracy.

In this study, we present a pioneering ML-based framework that leverages patient-level EHR data to achieve the following objectives:

1. Creating a comprehensive decision support tool that delivers five crucial diagnostic and prognostic predictions for various diseases, using COVID-19 as a test-case. These predictions encompass the likelihood of infection, ventilation requirements, mortality risk, duration of hospitalization, and days of intensive care unit (ICU) care needed, each of which were key dimensions for patient treatment for COVID-19.
2. Emphasizing the key factors influencing these diagnostic and prognostic outcomes, thus shedding light on critical determinants that could impact disease management and treatment planning.
3. Facilitating a deeper understanding of the intricate relationships among various factors and their influence on the disease outcome by identifying potential interaction effects.

December 31, 2019 marked the first official report of the novel coronavirus of 2019, or COVID-19, in Wuhan, China. Because of its unprecedented ability to transmit through multiple mechanisms (Kucharski et al. 2020), it rapidly spread globally, leaving governments and healthcare institutions scrambling to understand the disease and mitigate the spread. World Health Organization (WHO) declared COVID-19 a global pandemic in early March of 2020. The rapid transmission rates were exacerbated by the fact that 20% of those infected become asymptomatic carriers (Mallapaty 2020). The pandemic has had a severe impact on the global economy, with the United States gross domestic product (GDP) dropping 32.9% in the 2nd quarter of 2020, the worst drop on record.

The methodology we propose is flexible and generalizable, designed to accommodate diverse types of clinical data that can be extracted from EHRs. Our approach is particularly suitable for situations where datasets may contain missing information or require the integration of temporal data into a static predictive model. By focusing on the interpretability of model findings, we prioritize the identification of key factors and their interactions, which is crucial in healthcare applications where the rationale behind predictions is as important as the predictions themselves.

Our framework’s utility extends beyond the immediate context of COVID-19, offering potential applications for a broad range of diseases. The interaction maps and charts generated by our ML models serve as powerful exploratory tools that can reveal complex interdependencies between clinical, demographic, and potentially socioeconomic factors within the EHR data. These visualizations can readily suggest hypotheses regarding the mechanisms of disease progression, the impact of comorbidities, or the influence of treatment modalities on patient outcomes.

Additionally, we have demonstrated the application of our framework through a proof-of-concept decision support tool that provides real-time, data-driven predictions. This tool is designed to be integrated seamlessly into clinical workflows, enhancing patient care by supporting clinicians with actionable insights derived from ML predictions. In essence, our work showcases the potential of ML as a transformative tool for diagnostic and prognostic predictions, embodying the collective efforts toward improved healthcare outcomes and fostering opportunities for interdisciplinary research in health services methodology.

The remainder of the paper is structured as follows: Sect. 2 reviews prior literature and sets the context for our study by summarizing relevant ML applications in healthcare, particularly focusing on COVID-19 research. Section 3 details our methodology, including dataset and preprocessing steps, variable selection and importance, cross-validation and model training, performance metrics, and our novel approach to identifying interaction effects. The results of our study are presented in Sect. 4, where we discuss the prediction performance of our models, key factors influencing disease outcomes, and the relationships among these factors. Section 5 engages in a discussion on the generalizability of our framework to other diseases, its applications in clinical decision-making, and its potential for generating new research hypotheses. Finally, Sect. 6 concludes the paper with an overview of our contributions to health services and outcomes research methodology and the implications of our findings for healthcare practice and policy.

## 2 Prior literature

Machine learning has been used for medical diagnosis, prognosis and patient behavior prediction extensively over the last decade (Dolatsara et al. 2020; Misiunas et al. 2016; Mueller-Peltzer et al. 2020; Nasir et al. 2019, 2020; Piri 2020; Simsek et al. 2020; Topuz et al. 2018). However, given that the behavior of the underlying virus and its effects on humans are being observed for the first time, research has just started to come out on this topic. In addition to traditional medical and sociological research on the disease and its effects, ML methods are also being applied in various ways to data about the pandemic. Most of this research can be categorized into two categories: (1) predicting the spread of the disease (broad-level predictions or forecasting) (2) predicting disease detection and prognosis (patient-level predictions or diagnosis/prognosis).

Broad-level predictions (i.e., forecasts) are based on methodologies that use time-series data of the spread of the virus as well as broad sociological factors to predict the future spread of the virus or other future characteristics of the pandemic. These studies do not take into account data about individuals. Thus, these capture the broad trends that emerge from the complex interactions between the virus and human society, without looking at the underlying factors. Patient-level predictions (i.e., diagnosis/prognosis) use more granular data at the patient level to predict certain outcomes for each patient. These may have observations about patients at multiple times, however the key difference is that these models are not based on past-values to predict future values. Instead, they take into account factors such as sociodemographic, medical and other personal level data to predict the outcomes for each person individually.

These areas are still being explored and the literature is limited, however, in Table 1, we provide a brief overview of the extant work (Apostolopoulos and Mpesiana 2020; Ardakani et al. 2020; Arora et al. 2020; Lalmuanawma et al. 2020; Li et al. 2020; Ribeiro et al. 2020; Shi et al. 2020; Sun et al. 2020; Tuli et al. 2020; Vaid et al. 2020; Yadav et al. 2020; Yang et al. 2020). Interested readers can also refer to the various review studies that list additional similar or overlapping works (Lalmuanawma et al. 2020; Shi et al. 2020). As can be seen in Table 1, most existing studies have focused on time-series forecasting to predict the spread, peak and decline of cases and related factors. Another commonly studied topic is x-ray/CT scan-based infection detection using deep-learning models. Some studies also describe optimization techniques for peripheral problems to improve operational performance against COVID-19 (Santini 2021). However, the use of structured clinical and socioeconomic data, which in principle should provide useful information about the spread of the virus, has not been reported. We posit that the principal reason for this may be the lack of availability of reliable patient-level clinical data for COVID-19 cases.

As such, in this work, we use a large EHR dataset that was released by the Veteran Affairs’ Veteran Health Administration (VHA) Innovation Ecosystem and U.S. Food and Drug Administration’s precisionFDA to study the risk and protective factors of COVID-19 in the veteran population. The dataset was artificially generated by the VHA using Synthea (Walonoski et al. 2018), a synthetic patient EHR generation software that utilizes real disease models to produce synthetic anonymous EHR datasets. We use the dataset to model five patient outcomes: (1) infection likelihood, (2) death likelihood, (3) ventilation requirement likelihood, (4) hospitalization days needed, (5) days in ICU needed. For each of these outcomes, we use the ML models to identify factors important for this outcome and provide interaction maps for the factors involved. We also show how various factors affect the effects of other factors on the outcome.

## 3 Methodology

**Fig. 1:**
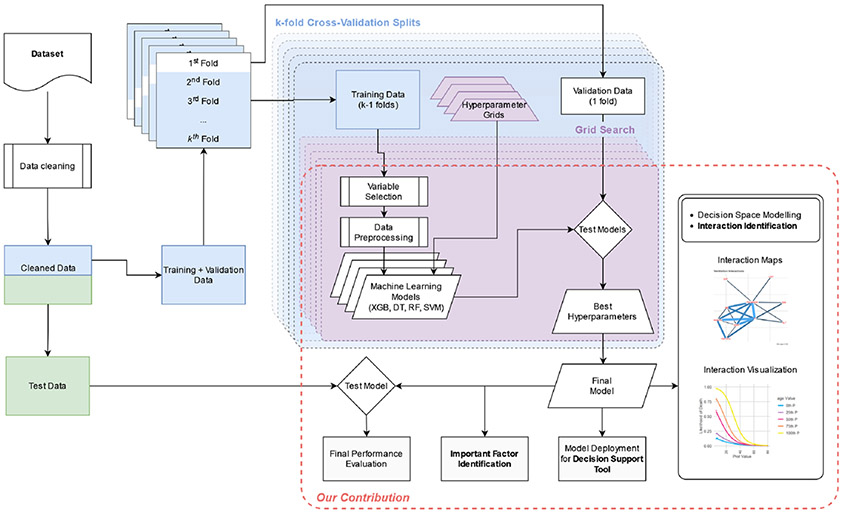
ML framework for each outcome proposed in this work

Figure 1 shows the proposed ML framework that enables (1) a methodology that can be used to remove problematic predictors to understand important predictors, (2) a novel exploratory methodology to identify potential interactions between the predictors that can be used to make informed policy decisions, and (3) a unique ML framework that proposes an end-to-end pre-to-post diagnostic testing methodology for COVID-19 that is useful for managers for tactical and strategic decision-making as well as for researchers and domain experts for novel interaction identification that can be used for generating new hypotheses. In the following sections, we describe the various components of this methodological pipeline.

### 3.1 Dataset and preprocessing

The dataset used in this work was released by the VHA Innovation Network in partnership with precision FDA for a competition to invite researchers to model the data in order to further the understanding of the disease (VHA Innovation Ecosystem and precisionFDA COVID-19 Risk Factor Modeling Challenge 2020). To protect patient identity, synthetic health records were generated using Synthea (Walonoski et al. 2018), a well-accepted method to generate research-ready anonymized data. Numerous studies show this method, as well as other synthetic methods, work as well as real data for large population sizes for the purposes of predictive analytics (Benaim et al. 2020; Chen et al. 2019; Gebert et al. 2018; Zhang et al. 2020).

Use of synthetic datasets in the healthcare ML community is common given the many barriers to quality data access (King et al.; Spasic and Nenadic 2020). In this case, the situation is worse given the high potential economic value of such data, thus resulting in complete unavailability of clinical or EHR datasets for COVID-19 patients. The VHA graciously provided this dataset, based on the vast population under their care, and this was made possibly only because of this anonymization.

The dataset consisted of 16 files structured in the standard EHR format, which are listed below. Data dictionaries for these files can be found on the Synthea Github wiki [34].

The dataset contained medical records for 117,959 patients. These records included lab tests, clinical observations, conditions, allergies, patient encounter data and more. It is important to note though that differing amounts of data were available for each patient. The data, as provided, was structured in the long format, i.e., one column lists the name of included variables, while another column lists their corresponding values.

In our study, we processed time-series observations from immunizations, conditions, allergies, and encounters tables by converting them into additional features within our primary analysis table, effectively transforming the dataset into a cross-sectional format. Specifically, for immunizations and conditions, we counted occurrences and used these counts as features, while for allergies and conditions currently affecting the patient at the time of COVID-19 testing, we encoded their presence as binary variables. By doing so, we collapsed the longitudinal data into a single row per patient, enabling the application of ML algorithms that require a fixed number of features for each instance. This conversion was paramount as it allowed us to include temporal clinical events as part of our cross-sectional predictive modeling framework, ensuring that each patient’s record reflected their medical history up to the point of COVID-19 testing without directly modeling the data as a time series.

A consequence of this process was that in case a given patient did not have a certain test or encounter in their records, the test would be marked as a missing value in the data. Given the large number of possible tests and encounters included in the dataset, this resulted in a large number of missing values in the dataset as not every patient has had every test and encounter. As such, for this analysis, an ML model that can inherently deal with missing values was used to reduce the complexity of the analysis. Additionally, for the prediagnostic prediction model (i.e., predicting infection), patient data up to the day before the patient presented themselves for a COVID-19 test was used. For postdiagnostic prediction models (i.e., the rest of the models), patient data up to and including but not exceeding the day of COVID-19 testing was used.

Data from selected tables shown above was combined into a single table with each row corresponding to each patient with their corresponding records and outcomes. After preprocessing, the dataset included 492 predictors and five outcome variables. However, the dataset had 63% missing values for the input predictors. These missing values only occurred in columns constructed from tables that had differing levels of information for different patients, i.e., in the observations.csv file, as shown in Table 2. For example, for allergies and conditions, a patient could be labelled to have an active allergy or condition if an active record of one exists. If no record exists in this case, an assumption of no allergy or condition can be made. However, in the case of medical tests, different patients have different medical tests on record, and no assumptions about missing tests can be made outright.

Since our dataset had 63% missing values, we used a tree-based models, i.e., extreme gradient boosting (XGB) Chen et al. (2015) and CART decision trees (DT) to model the various outcomes in the data. Furthermore, since support vector machines (SVM) and random forest (RF) do not work with missing values, data for these models was preprocessed to impute missing values with the median value for each variable.

Details about the outcome variables included in the dataset are provided in Table 3. Variable selection and variable importance criteria are detailed in the next section. A file with a complete list of the 492 predictors from the raw dataset (Synthea 2020) and their descriptions can be found in "Appendix A".

To ensure that our models provide good predictive performance using factors available at decision-time without future leakage, each of the models is trained based on related EHR data that is available up to a certain date, as shown in the last column of Table 3. For example, for the model prediction infection, the model is only trained and tested using EHR data available up to the day before the patient is presented for covid-19 testing.

### 3.2 Variable selection and importance

Based on the “splitting” criteria used by tree-based algorithms (refer to Chen, He [35]), one way to find the most important variables in such tree-based algorithm is to look at which variable was used to create the most splits in the learned model. The top 30 most important variables are reported for each outcome in this work.

Given the aforementioned “splitting” criteria used in XGB and other tree-based algorithms, it is important to note that such algorithms use the variables that create the “cleanest” splits for the outcome between the data at each node. Thus, at each split (i.e. node in a tree), the algorithm will randomly choose a subset of variables and within these, it will choose the variable that differentiate the data into the outcomes most efficiently. This highlights the fact that factors included in ML models highlight associations with the outcome only.

Another consequence of the above is that some variables may dominate variable importance in the learned model because it would be used for almost all splits in the algorithm. A very high importance can indicate one of only two things: (1) a very strong predictor (in the case of a simple X → Y mapping), or (2) a problematic variable (in the case where it is unlikely that X → Y is linked so strongly through a single variable). Given that our outcomes are highly complicated problems, it is unlikely that these outcomes can be mostly explained with a single variable. As an example, before variable selection, following were the top variables for each model.

In Table 4, only variables with a variable importance greater than 1% are shown. For the Infected model, three variables cumulatively contribute to 93.95% of the splits in the model. An XGB model trained on just these three variables has an AUC of 0.9811 and a sensitivity and specificity of 99.99% and 96.39% respectively. However, it is unlikely that our outcome can be explained to such an extent using just these three variables. Indeed, this can be confirmed when this simplified model is tested with the holdout set, where its prediction performance (AUC = 0.902, Sensitivity = 69.7%, Specificity = 99.6%) does not match the validation performance, an indication that the model does not generalize. The same principle is true for all the other models as well, as shown in Table 4.

As such, problematic variables were dropped from the analysis for that outcome since they may be exhibiting simultaneity problems with the outcome or causing the model to overfit on invalid patterns due to missing data. This was done using an iterative model training method:

1. Start with the complete dataset
2. Train model using dataset
3. Observe most important variable
4. If importance is very high, remove the variable from the dataset
5. Repeat from 2 until the highest importance variable, either:
  - Is below a threshold of 0.35 and the next highest importance variable is lower; or
  - Should remain in the dataset based on expert opinion

The precise cutoff for each model was chosen based on the biggest importance score drop achieved against the smallest AUC or RMSE drop, which also led to a reasonable final AUC. An example of the importance/AUC drop for the death outcome is shown in Table 5. The table shows the most important variable as well as performance metrics for XGB models iteratively trained using the above method. Note that each row signifies a model that was trained without the variables in the rows above it. In the case of this example, it can be seen that the model’s AUC does not change significantly down to the last rows, with row 11 showing the point chosen for the final model. As an example, the model in row 1 uses one variable for 93.9% of its splits, with a handful of other variables used for the remaining splits. As such, if a single factor explained COVID-19 death to such an extent, it would have been widely known by now.

Given computational constraints (model tuning and training with variable selection), the variable selection procedure was performed only with XGB, given XGB is expected to perform the best, and at a fraction of the training time of the remaining models.

### 3.3 Cross validation and model training

**Fig. 2:**
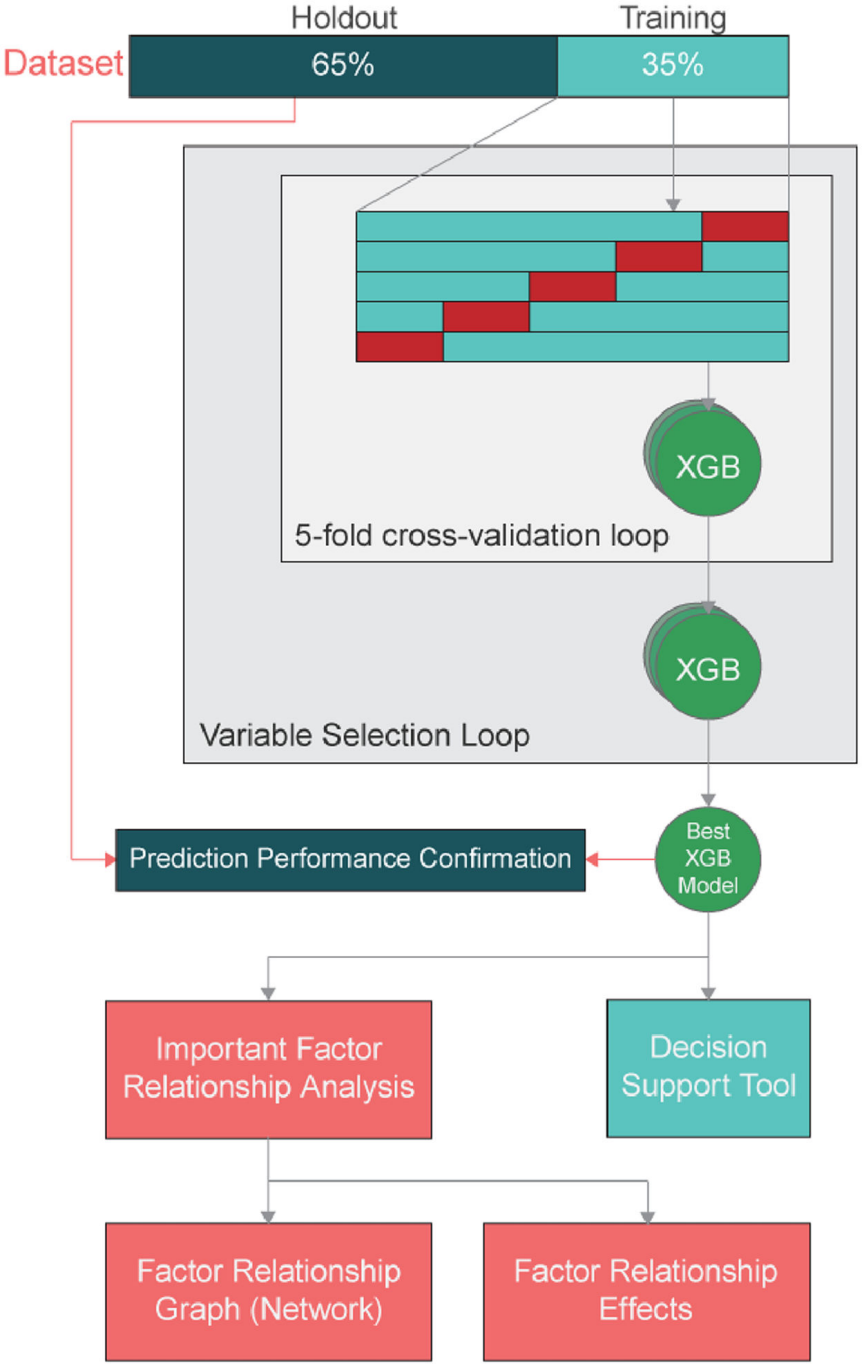
Nested cross-validated train pipeline

To ensure the validity of ML model, we use a nested cross validation technique as shown in Fig. 2. The larger the test set, the more confident we can be in the performance metrics reported by the model. If the model’s performance metrics for the test set (referred to as the holdout set in this work) are very close to the model’s performance for the training set, it indicates that the model is not over- or under-fitting the data and has correctly generalized the relationships present in the data.

We chose XGB because in applied ML problems, XGB is currently the best performing algorithm across all disciplines as is evidenced by leaderboards across all disciplines as well as its dominance in the academic literature in the past couple of years. XGB is resilient to overfitting (Chen et al. 2015) and can also inherently deal with missing values by imputing missing values at each split that minimize error at that split (Chen et al. 2015). Decision trees (DT) is one of the oldest and simplest learning algorithms, popular for its explainability. It works by splitting the data into segments successively, based on some splitting criteria, creating a tree structure that terminates based on a stopping criteria (Safavian and Landgrebe 1991). Support vector machines (SVM) are a popular kernel based learning algorithm that works on the basis of separating the data based on hyper-planes in a higher-dimensional space than the original data using the “kernel trick” (Osuna et al. 1997). Finally, random-forest (RF) is a widely-used tree-based learning algorithm that uses an ensemble of decision trees that are trained as weak learners in order to minimize over-fitting, a problem that individual decision trees are prone to (Breiman 2001). These remaining models were chosen to serve as a comparison against XGB. These models (DT, SVM and RF) took longer to train (by a factor of 10–1100 compared to XGB) and were therefore not used for our variable selection process. Detailed descriptions of these models are not included in this work, given length constraints. However, the interested reader can refer to (Boulesteix et al. 2012; Noble 2006; Song and Ying 2015) for more information about these models.

Based on preliminary analyses, using more than 30% of the data did not improve performance metrics of the ML models. This was ascertained by splitting the data into chunks of 5% and repeatedly training the ML models with additional chunks until the performance metrics stopped increasing. Therefore, for this work, 35% of the data was used for training and parameter tuning with fivefold cross-validation (Fushiki 2011), while the remaining 65% was used as the holdout set.

All ML models produce numeric outputs, both for classification or regression problems. For binary classification problems, ML models produce a continuous number between a range, usually 0–1, to signify a binary outcome, which is produced by comparing the continuous output against a threshold. A general starting point is to use 0.5 as a threshold; however, choosing 0.5 may lead to lower prediction performance in some metrics. As such, this threshold can be selected based on the receiver operator characteristic area under the curve (AUC) graph, to choose a point for best balance between accuracy for both classes (i.e., points closest to the top left corner of the graph), or to favor accuracy for either class, based on the requirements of a given problem. This type of threshold selection is called post-hoc threshold selection. In this work, we use post-hoc threshold selection for the classification models. The validity of each threshold is confirmed with the hold-out set.

Model parameters were tuned with a grid-search methodology to find the parameters that resulted in the best model performance. All tuning was done using fivefold cross-validation. Additionally, as can be seen in Table 1, the Death and Ventilation outcomes were severely imbalanced in the dataset. In our preliminary experiments, the model performance unfortunately did not improve with any synthetic generation algorithms like SMOTE (Chawla et al. 2002). The post-hoc threshold selection (Zhao 2008) was sufficient to provide a model that had balanced sensitivity vs. specificity, which was confirmed with the cross validation and subsequently the holdout sets.

### 3.4 Performance metrics

Since we have both classification and regression models in this work, we report the AUC for the classification models and the RMSE (root-mean-squared error), R^2^ and MAPE (mean average percentage error) for the regression models. The AUC is chosen for the classification models since it shows the models performance at all possible decision thresholds (i.e., all sensitivity/specificity pairs), however we also report the best sensitivity/specificity pair. The best pair from the holdout set is calculated based on threshold selection from the training set. Formulae for each of these measures are given below and Davis and Goadrich (2006) provide detailed explanations.


(1) $$Sensitivity=\frac{True\;positives}{Actual\;positives}$$

(2) $$Specificity=\frac{True\;nagatives}{Actual\;negatives}$$

(3) $$Precision=\frac{True\;positives}{Predicted\;positives}$$

(4) $$RMSE=\sqrt{{\left(predicted-observed\right)}^{2}}$$

(5) $$MAE=\frac{\sum_{t=1}^{n}|observed_{t}-predicted_{t}|}{n}$$

### 3.5 Interaction effects

Using a novel methodology (Nasir et al. 2021), the top 30 variables for each outcome’s model are also examined for possible interaction effects with other variables present in each respective model based on the best performing ML models, i.e., XGB, for each outcome. Since the ML models are non-parametric, it is impossible to directly see how the models make predictions (Guo et al. 2021). However, given the models are making good predictions based on the relationships they learnt from the training data, these relationships can reveal information about the phenomenon described by the data.

To find these relationships, we perform sensitivity analysis for all predictor pairs that include the top 30 important variables, which allows us to observe if and how one variable impacts the effect of another variable on a given outcome. This is done by changing this variable pair’s values while keeping all the other variables fixed at their means, while observing the output. Variable pairs that demonstrate an interaction are detected using this methodology. Variables that are observed to have a large effect on the outcome as well as an effect on the effects of other variables can be deemed to be highly important for the outcome. The detailed algorithm for this methodology is provided below.

1. Start with a dataset where a mapping between inputs (X’s) and an output (Y) exists.
2. Model this mapping using one or more ML models. We use five XGB models, each trained using four alternating folds out of five from the training dataset.
3. For each variable pair:
  3.1 Split the input domain into quintiles.
  3.2 With each model:
    3.2.1 Plug in each variable combination to map the variable pair’s behavior.
    3.2.2 Subtract the quintile mean from each quintile (line).
    3.2.3 Sum up the area between the resulting curves to get the effect “size”.
  3.3 With all the models’ resulting effect sizes, calculate the mean, standard deviation and coefficient of variation (CV) of the size.
4. Filter variable pairs based on mean and CV values, selecting variable pairs with large mean and small CV values.

## 4 Results

### 4.1 Prediction performance

All prediction models were tuned and trained using a fivefold cross-validation methodology. The cross-validation performance for each outcome along with salient tuning parameters chosen for the ML models for said outcome are shown in Tables 6 and 7 respectively. Note that, for the infection prediction model (i.e., the prediagnostic model), we use the complete dataset to train the model. For the postdiagnostic models (i.e., death, ventilation, days hospitalized, and days in ICU), only the 73,843 patients infected with COVID-19 in the dataset were used to train the models. Thus, the post-diagnostic prediction tests, as suggested by the name, are done for patients who have been diagnosed as infected with COVID-19.

XGB performed the best in all cases, getting the highest performance metrics across the board. Furthermore, across the outcomes, all models had good performance; however, the Days in ICU prediction model is an outlier in terms of performance. This is most likely because the model could not find all the necessary information or signals in the data to better model the regression outcome for this problem.

Once the best performing model was identified, we confirmed its performance metrics by testing it again with the holdout set. In this work, we use a test set that was larger in size than the dataset used to train the model, as described in Sect. 3.2. This decreases the chances of any random sampling effects in the data skewing the performance metrics in any direction. The holdout set prediction performance is reported in Table 8 below. As can be seen, each model demonstrates very similar prediction performance to the validation set. Thus, we can confidently say the models are not over/under-fitting the data and the underlying relationships modeled by the algorithm can elicit meaningful information about the modelled problem.

### 4.2 Key factors

**Fig. 3:**
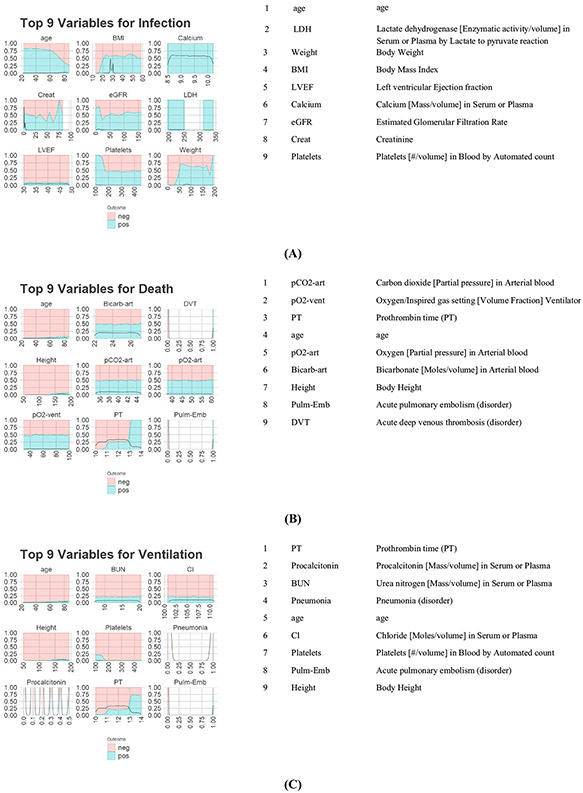

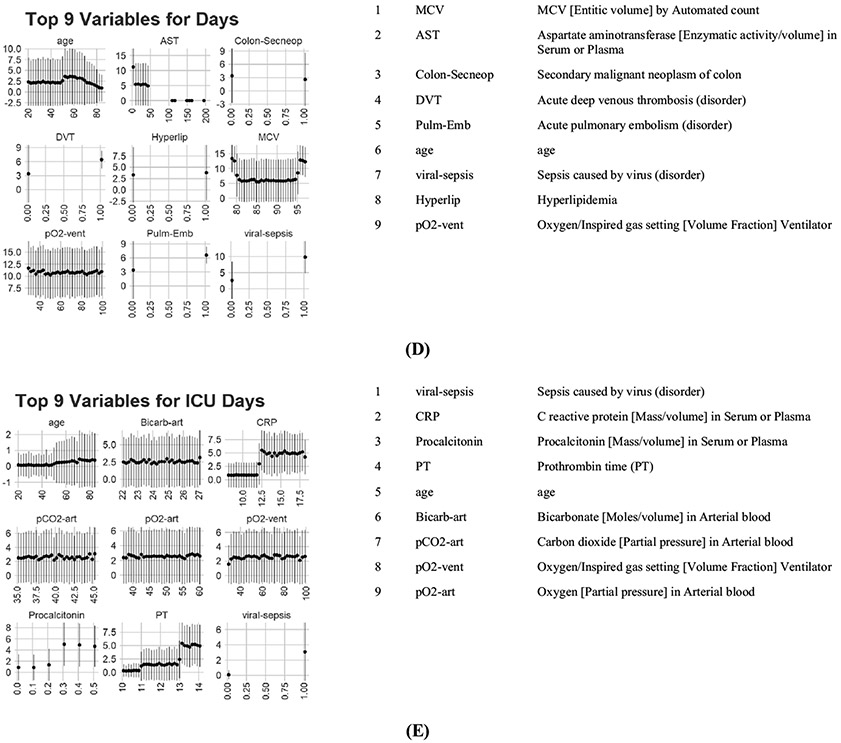
Important variables and their distributions

Figure 3A-E show the variable distributions as well as their descriptions. For the sake of brevity, we have limited the figure to 9 distribution graphs and their accompanying variable descriptions for each model; however, full lists of important variables can be furnished to interested readers upon request

The graphs for the classification outcomes show the density of each variable at a given value, as well as the proportion of outcomes (blue = positive, red = negative) found at that value. A blank portion on the graph signifies no observations found at those values. The graphs for the regression outcomes show the expected value for the outcome ± its standard deviation at any given value of the predictor.

It is interesting to note that for many variables, the outcome behavior does not seem to change in the dataset. However, since these are among the most important variables, this likely means that these variables provide good data separation in certain non-linear scenarios. We further explore these in the next section.

### 4.3 Relationships among key factor

**Fig. 4:**
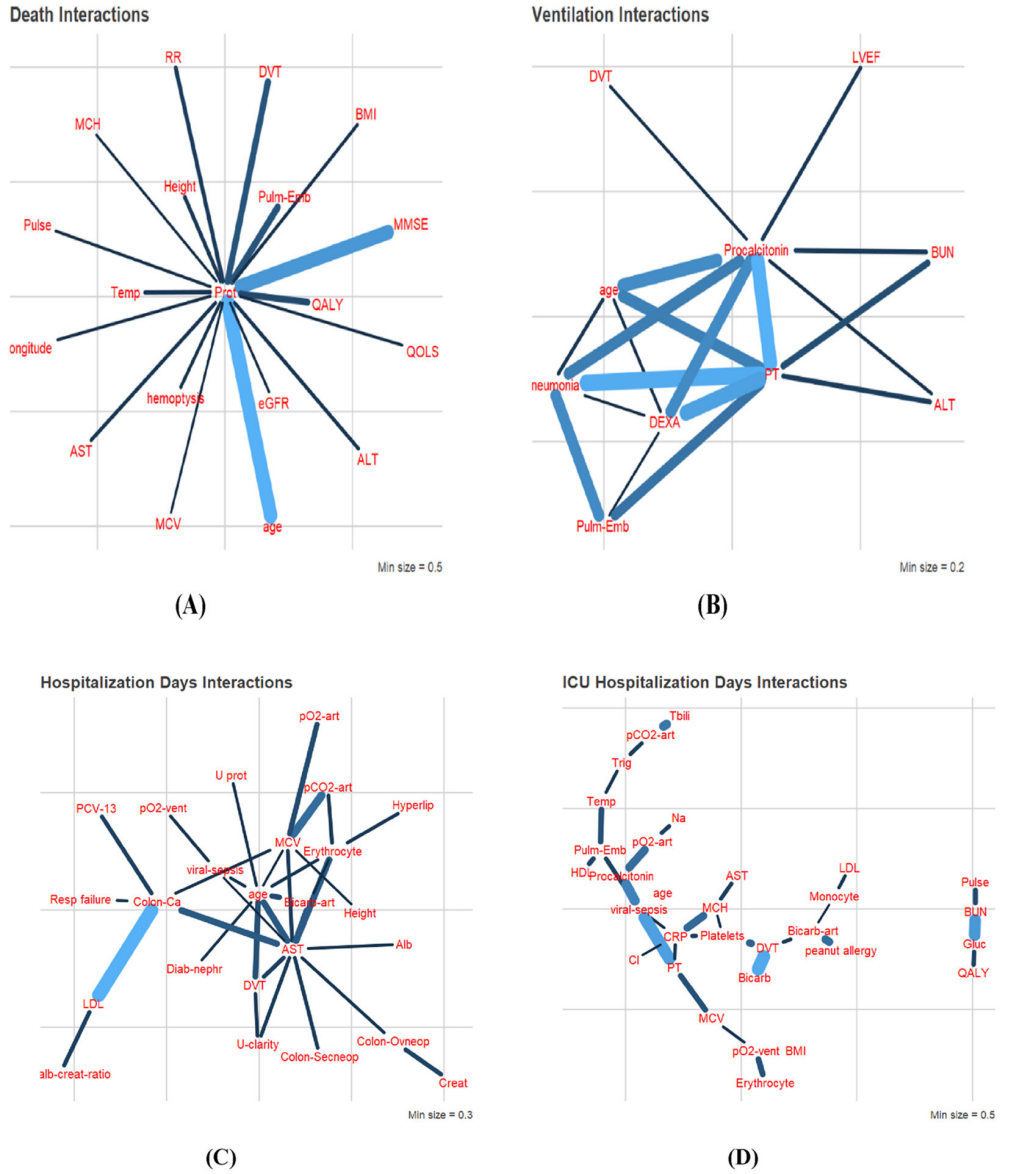
Relationship graphs for each outcome

Using the sensitivity analysis method described in Sect. 3.4, we obtain interaction relationships for each model, which are shown in Fig. 4. The infection model did not yield any significant interactions which likely indicate that different factors independently lead to certain people getting infected. It should also be noted that for the case of infection, exposure to the coronavirus is also an important factor that cannot be captured with this methodology using EHR data, despite controlling for location. Therefore, it is not surprising to find that the XGB model for infection does not exhibit any interactions between its predictors.

In Fig. 4, the edge thickness denotes the relative amount of change the variables show on the other’s effect within the variable pair. Highly connected variables are likely to play an important role in the outcome. Variables connected to highly connected variables may play a mediating role on the effects of many other variables.

**Fig. 5:**
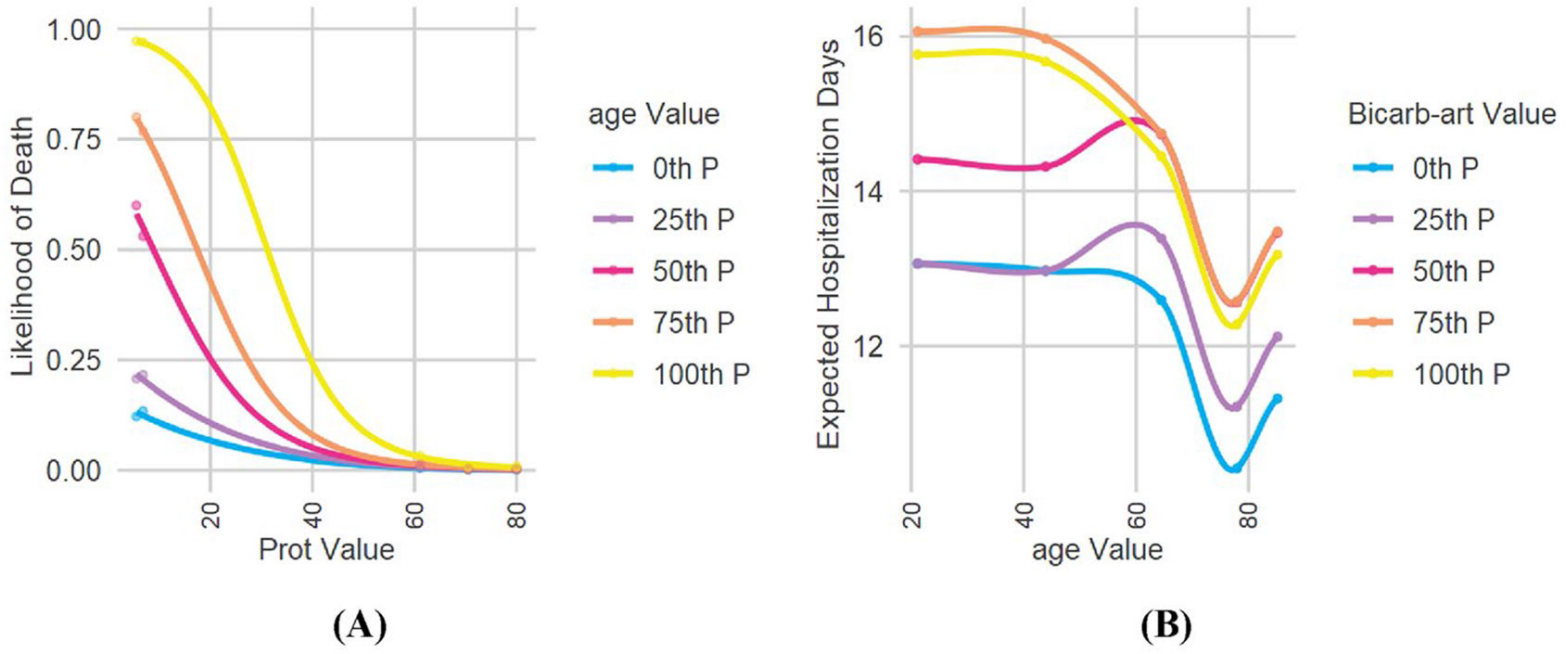
Select interaction charts; see text for description

Figure 5 shows two individual interactions for two different outcomes. Figure 4a shows that as age increases, the effect of variable “TEST_2885.2” or “protein in serum” increases, with lower values of protein in serum associated with a higher likelihood of death from complications of COVID-19. Similarly, Fig. 5b shows that the high values of “TEST_1960.4” or “bicarbonate in arterial blood” are associated with a longer hospitalization for COVID-19, an effect that decreases with increasing age, before increasing again. A complete list of observed interactions and their graphs can be provided to interested readers upon request.

## 5 Discussion

In this work, we have demonstrated that applying ML to EHR data can provide useful diagnostic and prognostic predictions for any given disease. The strength of our method is the multidimensional pre- and post-diagnostic outcome prediction framework that can work with any available information and is resilient to some missing data. We also devise a novel methodology with the ML models to further shed some light on how various predictors affect each other and the outcome by identifying potential interaction effects. This methodology is an example of explainable AI and allows us to pry into the blackbox ML model.

Based on the prediction models proposed here, for any given patient before and after diagnosis, decision-makers can augment their decision-making processes with the following predictions:

- **Before** diagnosis and initial testing
  - What is the likelihood of the patient being infected?
- **After** diagnosis and initial testing
  - What is the likelihood of the patient to require a ventilator?
  - What is the likelihood of the patient to die of complications?
  - How long is the patient likely to require hospitalization?
  - How long is the patient likely to need ICU care?

**Fig. 6:**
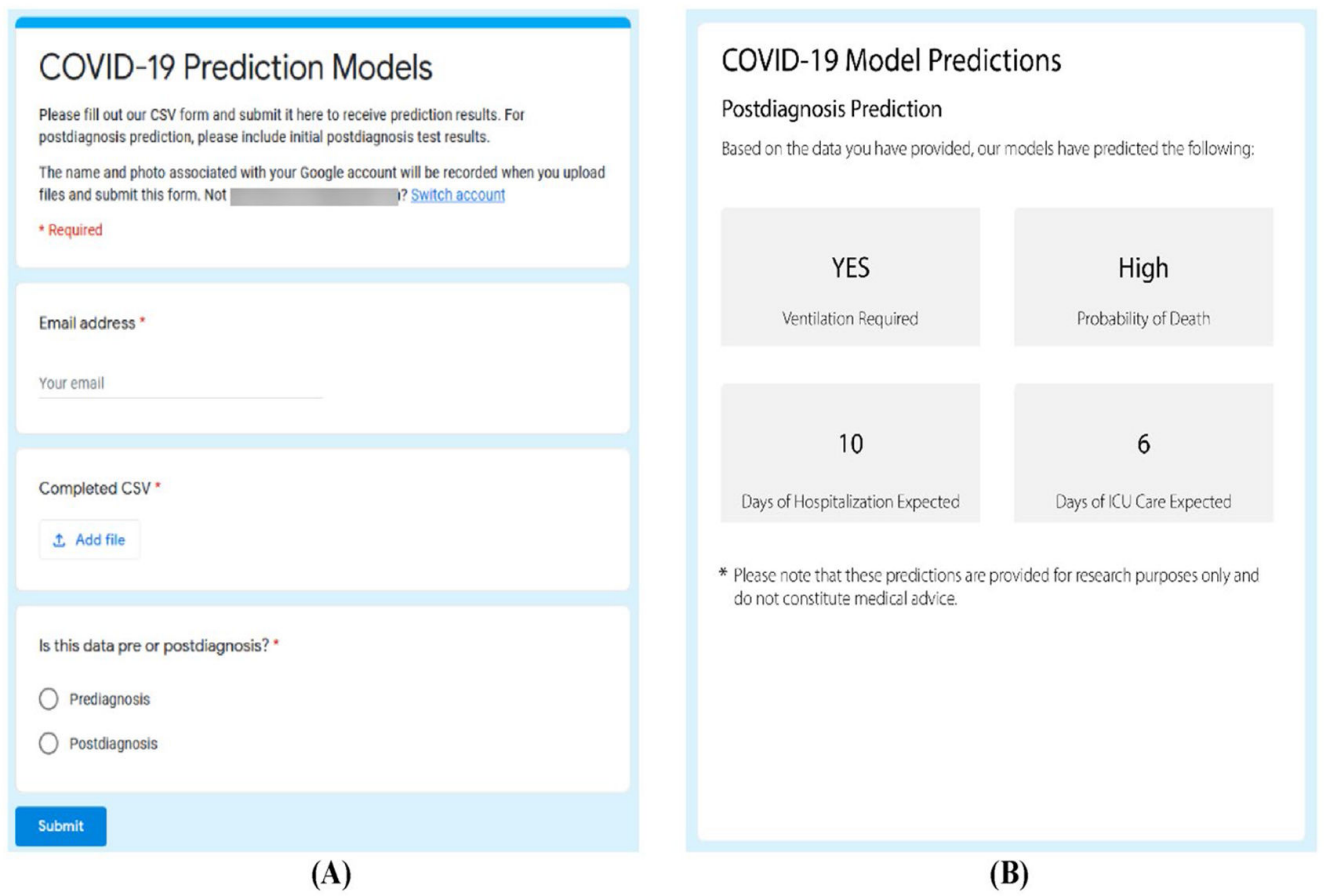
Decision support tool input form and example results

Combined, these predictions can provide a comprehensive picture of the level of care a given patient will need. Additionally, these predictions can be combined with optimization strategies as described in Azcarate et al. (2020), Li et al. (2021) to improve resource utilization and patient outcomes. As such, we have implemented an online decision support tool, linked in "Appendix A", to enable decision-makers to augment their available information with data-driven predictions models, as shown in Fig. 6. One key benefit of these models is that complete information is not required, so users need to only provide information about tests/conditions or other clinical data that is available. However, it is important to note that more information will allow for a more accurate prediction, and prediction accuracy will drop sharply for a given outcome if many of the important variables identified for said outcome are absent. In our dataset, on average, each patient has 37% variables populated with data, which yields the performance metrics reported in this paper.

In this work, we also provide interaction maps and charts for all post-diagnostic outcomes. Understanding these relationships and mechanisms with traditional domain-specific research methodologies can lead to new and potentially beneficial avenues for diagnosis and treatment.

### 5.1 Generalizability of the framework to other diseases

The framework we propose is fundamentally disease-agnostic. The methodology for data preprocessing, model training, variable importance assessments, and interaction effect analysis does not rely on disease-specific assumptions. Instead, it is designed to handle various types of clinical data that can be extracted from EHRs, including diagnoses, vital signs, laboratory results, and treatment information. This flexibility is critical in adapting the framework to other diseases.

To explain further, the generalization potential to other diseases can be supported by the following points:

1. *Data-Driven Approach:* Our ML models are trained on patient-level data, which includes a wide array of clinical parameters. This approach ensures that the models learn patterns that are predictive of the outcomes of interest, irrespective of the specific disease.
2. *Modular Framework for Hypothesis Generation:* The framework we have developed is modular and disease-agnostic, designed to facilitate the seamless integration of datasets for various diseases beyond COVID-19. Its consistent preprocessing and training pipelines ensure easy adaptability with minimal adjustments, making it a versatile tool for different healthcare contexts. The transferability of predictive patterns, such as the significance of laboratory values, vital signs, and comorbidities, underscores the framework’s utility across diverse diseases. Furthermore, our methodology for identifying key factors and their interaction effects is applicable irrespective of the disease under study. This capability provides critical insights for personalized medicine and targeted interventions, as it allows for the extraction of valuable information from the sensitivity analysis and interaction effect identification, which can be applied to enhance the understanding and treatment of other diseases.
3. *Explainable AI:* Our explainable AI approach can be particularly valuable for clinicians and researchers in understanding the underlying mechanisms of different diseases. The interaction maps generated by our framework can reveal complex relationships between clinical variables, offering hypotheses for further investigation.
4. *Proof-of-Concept Decision Support Tool:* The online decision support tool presented in this study, while demonstrated with COVID-19 data, can be adapted to other diseases. The interface and underlying predictive models can be updated with disease-specific datasets to provide real-time predictions for a wide range of health conditions.

### 5.2 Application of the framework in clinical decision-making

The framework can be used by practitioners in several ways to support decision-making.

#### 5.2.1 Risk stratification

Clinicians can use the framework to identify patients who are at an increased risk of experiencing adverse outcomes, such as severe complications or mortality. This risk stratification tool allows healthcare providers to tailor their approach, potentially opting for more aggressive treatment or closer monitoring for high-risk individuals.

#### 5.2.2 Personalized care plans

Predictions generated by our models can inform the development of personalized care plans by considering individual patient factors that significantly impact their prognosis. This helps ensure that treatment recommendations are tailored to the specific needs and risks associated with each patient.

#### 5.2.3 Decision support tool

We have developed an online decision support tool that integrates our framework, enabling clinicians to input patient data and potentially receive instant predictions regarding infection likelihood, ventilation requirements, mortality risk, hospitalization duration, and ICU care duration. This tool is designed to be user-friendly and to provide actionable insights that can guide immediate clinical decisions.

#### 5.2.4 Understanding interaction effects

The framework not only identifies key factors influencing health outcomes but also elucidates how these factors interact with each other. Understanding these complex relationships can aid clinicians in recognizing potential synergistic or antagonistic effects among patient characteristics, comorbidities, and other clinical parameters. This knowledge can drive more informed and nuanced decision-making. For example, understanding that a particular comorbidity may increase the risk of requiring intensive care for COVID-19 patients could prompt a clinician to monitor certain patients more closely or adjust their treatment plan accordingly.

### 5.3 Potential research hypothesis generation methodology

Researchers from various disciplines can use the framework and its outputs to explore hypotheses about disease mechanisms and the impact of various factors on health outcomes. The interaction maps generated by our ML models serve as a powerful exploratory tool that can unveil complex interdependencies between clinical, demographic, and possibly socioeconomic factors within the EHR data. These visualizations can readily suggest hypotheses regarding the mechanisms of disease progression, the impact of comorbidities, or the influence of treatment modalities on patient outcomes.

For example, epidemiologists could leverage these maps to hypothesize how environmental factors may synergize with genetic predispositions to affect disease susceptibility or severity. The interaction effects that we identify could prompt biostatisticians to consider novel statistical models to account for such complexities in their analyses. Health policy researchers might investigate the interplay between healthcare access and outcomes, thereby informing policy development and resource allocation strategies. Researchers interested in the social determinants of health could explore how different demographic factors intersect to influence health outcomes, potentially leading to more targeted public health interventions.

Furthermore, the adaptable nature of our decision-support tool offers a platform for researchers to test these hypotheses in practical, clinical settings. By inputting specific data points, they can observe how changes in one variable could affect the predictions for a patient’s outcome, thereby gaining insight into the potential causal pathways.

The framework provides a flexible foundation for various applications, from direct clinical support to exploratory research across healthcare and related fields. By adapting the framework to specific datasets and research questions, users can extract valuable insights to inform practice, policy, and further scientific inquiry. A limitation of this work is that it is based on synthetic data. However, this limitation was unavoidable since EHR or any clinical data for COVID-19 patients is completely unavailable to the wider research community (King et al.).

## 6 Conclusions

In conclusion, our study introduces a robust ML-based framework that leverages EHR data to deliver comprehensive diagnostic and prognostic predictions across a range of health outcomes. Using COVID-19 as a test case, we have demonstrated the framework’s ability to predict infection likelihood, mortality risk, ventilation requirements, duration of hospitalization, and ICU care needs.

Key contributions of our work include:

1. Multidimensional Outcome Prediction: Our framework provides a multidimensional perspective on patient prognostication, enabling healthcare practitioners to make informed decisions at various stages of patient care, from initial diagnosis to critical post-diagnostic care decisions.
2. Identification of Key Factors: Through the application of ML algorithms, we successfully identify key factors that influence disease outcomes. These factors provide valuable insights into the determinants of disease progression and patient response to treatment.
3. Interaction Effect Analysis: A novel aspect of our study is the exploration of interaction effects among predictors, offering a deeper understanding of the complex relationships that exist within clinical data. This exploration is facilitated by our sensitivity analysis methodology, which is a significant step toward explainable AI in healthcare.
4. Framework Generalizability: While our study focuses on COVID-19, we emphasize that the proposed framework is disease-agnostic and can be adapted for predictive modeling in a variety of diseases beyond the current pandemic, showcasing its broad applicability and potential for wide-ranging impact in healthcare.
5. Practical Decision Support Tool: We present a proof-of-concept decision support tool that encapsulates our ML models, allowing for real-time, data-driven predictions to augment clinical decision-making processes. This user-friendly tool can be easily incorporated into clinical workflows to improve patient care.
6. Research and Policy Implications: Our interaction maps and charts provide a resource for researchers to generate hypotheses and explore complex disease mechanisms. These visualizations can guide further investigation and inform health policy development, particularly in understanding the effects of interventions and healthcare services on patient outcomes.

The proposed framework is a powerful tool for healthcare practitioners and decision-makers to augment their decision-making processes with real-time data-driven predictions. In the case of this work, we demonstrated our framework with a COVID-19 dataset, that included various COVID-19 prognoses such as death, ventilation, days hospitalized, and days in ICU. The methodology was applied to a large dataset of EHRs and was shown to be effective in predicting all five outcomes. In addition, we also presented a novel methodology to identify potential interaction effects between the predictors, which can provide further insights into the factors that influence the outcomes of the disease.

The results of this study suggest that ML can be a valuable tool for predicting the prognostic and diagnostic factors of any disease. Such ML model tools can be used to improve patient care by helping clinicians make more informed decisions about treatment. Additionally, the identified interaction effects can be used to develop targeted interventions that can help to reduce the severity of the disease. The methodology presented in this paper is generalizable to any disease and can be used with any underlying ML algorithms. This makes it a valuable tool for healthcare providers and researchers who are interested in improving the outcomes of disease.

Our research offers a significant advancement in health services and outcomes research methodology by employing ML to extract valuable insights from complex clinical datasets. The methodology we propose is a powerful tool for enhancing healthcare quality through improved decision-making and has the potential to catalyze research across multiple disciplines within the healthcare domain.

## Supplementary Material

Supplementary Material

## Figures and Tables

**Table 1:** Select extant literature

Study	Data	Models	Findings	Type
Tuli et al. (2020)	Our World in Data COVID-19 Dataset	“Robust Weibull fitting” model	The authors show a 47.53 MAPE against 63.36 MAPE of a baseline gaussian model for worldwide cases. Also provide countrywise predictions and performance metrics	Forecast
Vaid et al. (2020)	COVID-19 Data Repository by the Johns Hopkins University Center for Systems Science and Engineering	Unbiased hierarchical Bayesian estimator approach to infer past infections from current fatalities	The authors used current deaths to infer past actual (apparent + hidden) coronavirus spread. Two approaches yielded the same conclusions: The US and Canada may have had between 1.5 and 2 times the reported number of infections	Forecast
Yadav et al. (2020)	WHO COVID-19 case statistics	Support vector regression	The authors use SVM to forecast the spread, mitigation and recovery for COVID-19 for multiple countries	Forecast
Ribeiro et al. (2020)	Brazilian State Health Offices	ARIMA, CUBIST, RF, RIDGE, SVR	The authors try various time-series regression techniques for one- to six-step ahead regression to predict COVID-19 spread. Their best model was SVR with holdout errors less than 6.9%	Forecast
Yang et al. (2020)	Daily COVID-19 outbreak numbers for China, augmented with the 2003 SARS epidemic statistics	LSTM model, SEIR model	The authors created a Susceptible-Exposed-Infectious-Removed (SEIR) model along with an LSTM model to predict infection spread, peak and regression. They found the LSTM to have the best fit with actual observed data	Forecast
Arora et al. (2020)	Indian Ministry of Health and Family Welfare	Convolutional, stacked and bidirectional LSTMs	The authors use LSTMs to forecast the spread of COVID-19 in various Indian regions at the daily and weekly levels. They showed on average a 3.22% mean average percentage error across different Indian regions	Forecast
Apostolopoulos and Mpesiana (2020)	2 datasets comprising of x-ray images of confirmed COVID-19 cases in addition to healthy as well as bacterial and viral pneumonia x-rays	Convolutional neural networks (CNNs)	CNNs were trained using COVID-19 positive patient x-rays as well as control x-rays of healthy patients and bacterial as well as viral infections. The authors showed sensitivity (specificity) of 98.66% (96.46%)	Diagnose
Li et al. (2020)	4356 CT scans from 3322 patients	Deep neural net	The deep learning model used volumetric chest CT scans to detect COVID-19. The model had a 90% sensitivity and 96% specificity on the holdout set	Diagnose
Ardakani et al. (2020)	1020 CT image patches (510 positive, 510 negative)	Deep neural net	Deep learning models were used with images to detect COVID-19 infections. The best model showed an AUC of 0.994 on a holdout set	Diagnose
Sun et al. (2020)	336 cases from Shanghai	Support vector machine	Various clinical and demographic data about the participants was collected and used with the ML model to predict symptom severity. The best model had an AUC of 0.9757 on a holdout set	Diagnose

**Table 2:** File structure of dataset used

File	Description	Included in Analysis	Created missing values
allergies.csv	Patient allergy data	Yes	No
conditions.csv	Patient conditions or diagnoses	Yes	No
encounters.csv	Patient encounter data	Yes	No
immunizations.csv	Patient immunization data	Yes	No
patients.csv	Patient demographic data	Yes	No
observations.csv	Patient observations including vital signs and lab reports	Yes	Yes
careplans.csv	Patient care plan data, including goals	No	–
devices.csv	Patient-affixed permanent and semi-permanent devices	No	–
imaging_studies.csv	Patient imaging metadata	No	–
medications.csv	Patient medication data	No	–
organizations.csv	Provider organizations including hospitals	No	–
payer_transitions.csv	Payer Transition data (i.e. changes in health insurance)	No	–
payers.csv	Payer organization data	No	–
procedures.csv	Patient procedure data including surgeries	No	–
providers.csv	Clinicians that provide patient care	No	–
supplies.csv	Supplies used in the provision of care	No	–

**Table 3:** Outcome descriptions

Outcome	Problem Type	Description	Distribution (Whole data set)	Includes EHR Up to:
Infected	Classification	Did the patient test positive for COVID-19?	Yes: 73,843 (62.60%) No: 44,116 (37.40%)	Day before COVID-19 diagnosis
Death	Classification	Did the patient die due to COVID-19?	Yes: 5,325 (4.51%) No: 112,634 (95.49%)	Day of COVID-19 diagnosis
Ventilation	Classification	Did the patient require ventilation due to COVID-19?	Yes: 4,210 (3.57%) No: 113,749 (96.43%)	Day of COVID-19 diagnosis
Days	Regression	How many days did the patient need to be hospitalized for COVID-19?	Mean: 2.183	Day of COVID-19 diagnosis
Days in ICU	Regression	How many days did the patient spend in the ICU for COVID-19?	Mean: 0.263	Day of COVID-19 diagnosis

**Table 4:** Variable importance without problematic variable removal

XGB Model	Variable	Importance (%)	Cumulative (%)
Infected	Acute bronchitis (disorder)	76.03	76.03
	Concussion with loss of consciousness	12.78	88.81
	Sprain of ankle	5.14	93.95
Death	Lactate dehydrogenase [Enzymatic activity/volume] in Serum or Plasma	93.86	93.86
	QALY	0.72	94.58
Days Hospitalized	Lactate dehydrogenase [Enzymatic activity/volume] in Serum or Plasma	71.54	71.54
	Ferritin [Mass/volume] in Serum or Plasma	16.36	87.90
	Troponin I.cardiac [Mass/volume] in Serum or Plasma by High sensitivity method	4.36	92.26
	INR in Platelet poor plasma by Coagulation assay	4.19	96.45
Days in ICU	Lactate dehydrogenase [Enzymatic activity/volume] in Serum or Plasma	61.75	61.75
	INR in Platelet poor plasma by Coagulation assay	3.87	65.62
	Ferritin [Mass/volume] in Serum or Plasma	1.72	67.34
Ventilation	Lactate dehydrogenase [Enzymatic activity/volume] in Serum or Plasma	60.37	60.37
	Acute respiratory failure (disorder)	21.74	82.11
	Ferritin [Mass/volume] in Serum or Plasma	5.38	87.49
	Oxygen/Inspired gas setting [Volume Fraction] Ventilator	1.45	88.94
	Oxygen [Partial pressure] in Arterial blood	1.34	90.27

**Table 5:** Dominating variable behavior against model accuracy

#	Most important variable	AUC	Sensitivity	Specificity	Importance
1	LDH	0.9972	0.9699	1.0000	0.9386
2	Ferritin	0.9972	0.9699	1.0000	0.9387
3	D-dimer	0.9972	0.9699	1.0000	0.9386
4	Lymph	0.9972	0.9699	1.0000	0.9386
5	Troponin	0.9972	0.9699	1.0000	0.9386
6	INR	0.9973	0.9699	0.9994	0.9134
7	CK	0.9962	0.9667	0.9961	0.7274
8	CRP	0.9961	0.9656	0.9896	0.3947
9	viral-sepsis	0.9952	0.9614	0.9890	0.5867
10	Procalcitonin	0.9930	0.9603	0.9699	0.3072
11	pCO2-art	0.9911	0.9571	0.9677	0.2117

**Table 6:** Cross-validation performance for different models

Outcome	Model	AUC	Specificity	Sensitivity	Precision
Infected	XGB	**0.916**	**0.784**	**0.868**	**0.87**
	DT	0.8069	0.741	0.794	0.836
	RF	0.9051	0.775	0.858	0.864
	SVM	0.845	0.733	0.809	0.835
Death	XGB	**0.991**	**0.957**	**0.968**	**0.997**
	DT	0.969	0.951	0.967	0.995
	RF	0.9855	0.954	0.954	0.987
	SVM	0.964	0.955	0.958	0.988
Ventilation	XGB	**0.975**	**0.961**	0.879	**0.989**
	DT	0.955	0.901	**0.886**	0.947
	RF	0.971	0.958	0.876	0.964
	SVM	0.961	0.935	0.871	0.928

Outcome	Model	RMSE	R^2^	MAE
Days Hospitalized	XGB	**2.085**	**0.84**	**0.75**
	DT	3.365	0.583	1.568
	RF	2.236	0.821	0.932
	SVM	3.124	0.817	0.985
Days in ICU	XGB	**0.984**	**0.467**	0.246
	DT	0.993	0.455	**0.239**
	RF	0.989	0.461	0.257
	SVM	0.991	0.459	0.258

Bold indicates the best values for each case

**Table 7:** Model training hyperparameters

Outcome	Model	Hyperparameters
Infected	XGB	nrounds = 50, max_depth = 10, eta = 0.15, gamma = 0
	DT	cp = .002
	RF	mtry = 16
	SVM	sigma = 2.44 × 10–4, C = 8
Death	XGB	nrounds = 100, max_depth = 8, eta = 0.15, gamma = 0
	DT	cp = .05
	RF	mtry = 16
	SVM	sigma = 1.25 × 10–1, C = 8
Ventilation	XGB	nrounds = 150, max_depth = 2, eta = 0.15, gamma = 0
	DT	cp = .02
	RF	mtry = 64
	SVM	sigma = 1.56 × 10–2, C = 64
Days Hospitalized	XGB	nrounds = 50, max_depth = 6, eta = 0.15, gamma = 0
	DT	cp = .002
	RF	mtry = 32
	SVM	sigma = 2.44 × 10–4, C = 32
Days in ICU	XGB	nrounds = 50, max_depth = 6, eta = 0.15, gamma = 0
	DT	cp = .05
	RF	mtry = 16
	SVM	sigma = 2.44 × 10–4, C = 8

**Table 8:** Holdout set performance metrics for top-two best performing models

Outcome	Model	Specificity	Sensitivity	Precision
Infected	XGB	0.785	0.871	0.785
	RF	0.764	0.860	0.792
Death	XGB	0.968	0.956	0.998
	RF	0.957	0.948	0.990
Ventilation	XGB	0.879	0.959	0.998
	RF	0.881	0.943	0.974
XGB	Model	RMSE	R2	MAE
Days hospitalized	XGB	2.107	0.835	0.752
	RF	2.215	0.820	0.946
Days in ICU	XGB	1.013	0.469	0.257
	RF	1.014	0.464	0.256
