# Supplementary material for: A machine learning approach for diagnostic and prognostic predictions, key risk factors and interactions
Source: Health Serv Outcomes Res Methodol. Author manuscript; Available in PMC 2025 Mar 6. (PMC11884741; doi:10.1007/s10742-024-00324-7)
Supplement: Supplementary Material [file NIHMS1997091-supplement-Supplementary_Material.docx]

**Article Title:** A Machine Learning Approach for Diagnostic and Prognostic Predictions, Key Risk Factors & Interactions

**Journal:** Health Services and Outcomes Research Methodology

**Authors:** Murtaza Nasir*, Nichalin S. Summerfield, Stephanie Carreiro, Daniel Berlowitz,

Asil Oztekin

**Corresponding Author:**

Murtaza Nasir, Ph.D.

Assistant Professor,

Barton School of Business, Wichita State University

1845 Fairmount St Wichita, KS 67260, USA

Phone: 605-202-1804

**Email Address:** [academic@murtaza.cc](mailto:academic@murtaza.cc)

Appendix A – Supplemental Files

**Complete variable list with descriptions:**<https://www.dropbox.com/s/3anmw17eh8uss3t/varlist.csv>

**Online Decision Support Tool Proof of Concept:**<https://forms.gle/kLWsYRzPWpc8iCXw6>
